# Association of LAMA3 expression with perineural invasion and chemoresistance in pancreatic ductal adenocarcinoma

**DOI:** 10.1007/s12672-025-03971-5

**Published:** 2025-11-20

**Authors:** Yang Yang, Yongqiang Chen, Xiaohong Wang, Qikai Sun, Yeben Qian

**Affiliations:** 1 Department of Hepatobiliary and Pancreatic Surgery, The First Affiliated Hospital of Anhui Medical University, Hefei, 230022 Anhui China; 2 Department of Intensive Care Medicine, The First Affiliated Hospital of Anhui Medical University, North District, Hefei, 230022 Anhui China

**Keywords:** Pancreatic ductal adenocarcinoma, LAMA3, Nerve invasion

## Abstract

**Background:**

Pancreatic ductal adenocarcinoma (PDAC) is a common type of malignant tumor of the pancreas with high aggressiveness and low prognosis. Due to the insidious early symptoms of pancreatic adenocarcinoma, patients are mostly diagnosed at advanced stages with a high incidence of nerve invasion. With the rapid development of precision medicine, studying the molecular mechanisms behind PDAC can help its diagnosis and treatment, which is conducive to improving the prognosis of PDAC patients.

**Objective:**

To explore the correlation between LAMA3 expression and nerve invasion in pancreatic ductal adenocarcinoma tissues.

**Methods:**

Ninety-four patients with pathologically confirmed diagnosis of PDAC in the Department of Hepatobiliary and Pancreatic Surgery of the First Affiliated Hospital of Anhui Medical University were retrospectively collected from January 2023 to December 2023, and the patients’ clinicopathological data were collected and followed up for 5 months. Immunohistochemical staining was applied to detect the expression level of LAMA3 in cancer tissues, and paraneoplastic tissues were used as controls to compare the differences in the expression level of LAMA3. The Kaplan-Meier method was used to draw the survival curves, and the Cox proportional risk regression model was set up to analyze the correlation between the expression of LAMA3 and the nerve invasion.

**Results:**

Immunohistochemical staining results showed that LAMA3 was mainly expressed in the cytoplasm and appeared as yellow to brown granules.The positive expression rate of LAMA3 in PDAC cancer tissues was 63.83% (60/94), which was significantly higher than that in paracancerous tissues (12.77%, 12/84), and the difference between the two groups was statistically significant (*x*^*2*^ = 51.862, *P* < 0.001). Patients were categorized into nerve invasion negative (*n* = 30) and nerve invasion positive (*n* = 64) according to the presence or absence of nerve invasion.Cox proportional analysis regression results showed that the LAMA3 expression level was an independent risk factor affecting the occurrence of nerve invasion in PDAC patients. Survival analysis showed that median OS was significantly lower in patients with high LAMA3 expression and development of vascular invasion than in patients with low LAMA3 expression and no vascular invasion (*P* < 0.001); in TNM staging, median OS was significantly lower in patients with stage II than in patients with stage I (*P* < 0.001).

**Conclusion:**

LAMA3 expression level is an independent risk factor for the occurrence of neuroinvasion in PDAC patients; LAMA3 expression level, TNM staging and prognosis of PDAC patients are correlated; LAMA3 expression level may serve as a valuable biomarker for the occurrence, development, and prediction of prognosis of patients with PDAC, and also as a potential therapeutic target for PDAC patients.

## Introduction

 Pancreatic ductal adenocarcinoma (PDAC) is the most common histological type of pancreatic cancer, accounting for more than 90% of cases, and remains one of the most lethal malignancies, with a 5-year survival rate below 10% despite advances in therapy [1–6]. Early diagnosis is challenging, and most patients present with advanced disease, underscoring the need for reliable prognostic biomarkers.Perineural invasion (PNI), defined as tumor cell infiltration around or within nerve fibers, is a frequent pathological feature of PDAC and has been strongly associated with recurrence and poor prognosis [7–9]. However, sensitive molecular indicators for predicting PNI are still lacking.Laminin subunit alpha 3 (LAMA3), a critical component of laminin-332, plays an important role in cell adhesion, migration, and invasion [10–12]. Abnormal expression of LAMA3 has been reported in breast, ovarian, gastric, hepatic, and pancreatic cancers, and is associated with tumor aggressiveness [13–15]. Nevertheless, its role in PDAC, particularly in relation to PNI, remains unclear. Therefore, this study aimed to investigate the association between LAMA3 expression and PNI in PDAC and to evaluate its potential prognostic significance.

## Objects and methods

### Objects

Ninety-four patients with pathologically confirmed diagnosis of PDAC in the Department of Hepatobiliary and Pancreatic Surgery of the First Affiliated Hospital of Anhui Medical University (AMU) were retrospectively collected from January 2023 to February 2024, and the clinicopathologic data of the patients were collected and followed up. Inclusion criteria: (1) age > 18 years; (2) diagnosed as pancreatic ductal adenocarcinoma after pathologic confirmation; (3) complete patient history and clinical data; (4) patients signed informed consent. Exclusion criteria: (1) Combination of other malignant tumors and metastatic cancers; (2) Combination of severe cardiac, hepatic and renal insufficiency or other major systemic diseases; (3) Pregnant or breastfeeding women; (4) History of severe infections or use of immunosuppressants in the last six months. This study fully safeguarded the rights and interests of participating patients and was approved by the Ethics Committee of our hospital.

### Methodology

#### Clinicopathologic features

Clinicopathological characteristics of all patients were retrospectively analyzed, including gender, age, Body Mass Index (BMI), Carcinoembryonic Antigen (CEA), Cancer Antigen 19 − 9 (CA19-9), tumor size, tumor location, tumor differentiation degree, lymph node metastasis, vascular invasion, TNM stage, and medical history. Follow-up was performed for 5 months in a manner that patients were followed up by telephone every 1 month. The cut-off date for patient follow-up was January 31, 2024, and survival was calculated from the date of diagnosis to the cut-off date for follow-up, or until the date of death due to recurrence or metastasis.

#### Immunohistochemistry

Immunohistochemical staining (IHC) was used to detect the expression of LAMA3 in pancreatic ductal adenocarcinoma tissues and paracancerous tissues. All samples were fixed in 10% neutral buffered formaldehyde and embedded in paraffin, and prepared as 4µ m thick serial sections. The sections were deparaffinized and rehydrated by sequentially passing through xylene and a gradient ethanol series for deparaffinization and hydration. Antigen repair was performed in a microwave oven using sodium citrate buffer (PH 6.0) to expose the antigenic epitopes of LAMA3. To block endogenous peroxidase activity, sections were incubated in 3% hydrogen peroxide for 10 min. sections were incubated in a blocking solution containing 10% goat serum for 30 min to reduce nonspecific binding. During immunohistochemical staining, a LAMA3-specific primary antibody (e.g., rabbit anti-LAMA3 polyclonal antibody) was used, and the sections were incubated overnight at 4 °C at a dilution ratio of 1:100. On the following day, sections were washed three times for 5 min each with phosphate buffer solution (PBS). biotinylated secondary antibodies (e.g., goat anti-rabbit IgG secondary antibody) were used and incubated for 30 min at room temperature and washed again with PBS. Sections were incubated with streptavidin-peroxidase complex (SABC) for 30 min at room temperature and washed three times with PBS. For color development, 3,3’-diaminobenzidine (DAB) was used for the chromogenic reaction until a brownish-yellow precipitate appeared. At the end of the reaction, the sections were well rinsed with tap water, followed by light re-staining of cell nuclei with hematoxylin, and dehydrated and transparent through gradient ethanol and xylene, and finally sealed. When the immunohistochemical staining results were observed and evaluated under the microscope, the positive expression of LAMA3 was mainly localized in the cytoplasm and cell membranes, showing brownish yellow staining. To quantify the expression level of LAMA3, we used a semi-quantitative scoring system that combines the staining intensity and the proportion of positive cells for assessment. Staining intensity was categorized as 0 (no staining), 1 (weak staining), 2 (moderate staining), and 3 (strong staining); the proportion of positive cells was categorized as 0 (0–5%), 1 (6–25%), 2 (26–50%), 3 (51–75%), and 4 (76–100%). The final score was the product of the intensity of staining and the percentage of positive cells score, with a maximum score of 12. Two experienced pathologists independently evaluated the slides in a blinded manner. Inter-rater agreement was assessed using the kappa statistic, which showed good consistency (κ = 0.82). Discrepant cases were reviewed jointly to reach consensus.

### Statistical methods

Using SPSS 26.0 statistical software, the chi-square test was used for comparison of categorical variables, the rank sum test for comparison of ordered variables, and the two independent samples t-test for comparison of continuous variables. Survival analysis was performed using the Kaplan-Meier method to draw survival curves, and the Log-rank test was used to assess whether the differences were statistically significant.To investigate the independent risk factors affecting perineural invasion in patients with PDAC, a Cox proportional hazards regression model was established. Variables with *P* < 0.1 in univariate analysis were included in the multivariate Cox model. The proportional hazards assumption was tested using Schoenfeld residuals, and no significant violations were observed. Indicators that remained statistically significant in the multivariate model were considered independent risk factors. A P value < 0.05 was considered statistically significant.

## Results

### General clinical data of PDAC patients

In this study, we collected clinical data from 94 patients with PDAC, 61 male patients (64.89%) and female patients (35.11%), with the age of onset ranging from 39 to 83 years old, the median age of onset being 61 years old, 38 patients with Stage I (40.43%), 16 patients with Stage II (17.02%), 19 patients with Stage III (20.21%), 21 patients with Stage IV (22.34%), with a disease duration of 6–17 months, median duration of 11 months, and patient follow-up of 5 months. See Table 1.

### Expression of LAMA3 in PDAC cancer tissues and paracancerous tissues

**Fig. 1:**
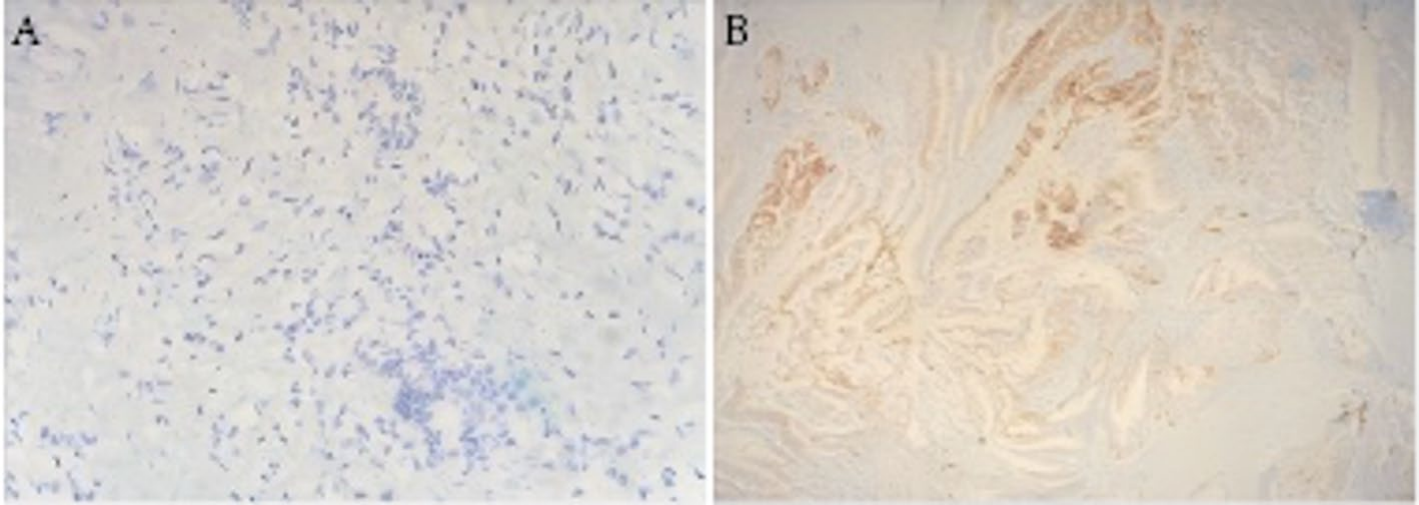
Immunohistochemical (IHC) staining of LAMA3 expression in paracancerous and cancerous pancreatic tissues. (A) Paracancerous tissue showing low LAMA3 expression (×200). (B) Cancerous tissue showing high LAMA3 expression (×200). Positive staining was defined as brownish-yellow coloration in the cytoplasm and/or cell membrane. IHC scores were calculated as the product of staining intensity (0–3) and percentage of positive cells (0–4), with a maximum score of 12

Immunohistochemical staining results showed that LAMA3 was mainly expressed in the cytoplasm as yellow to tan granules.The positive expression rate of LAMA3 in PDAC cancer tissues was 63.83% (60/94), which was significantly higher than that in paracancerous tissues (12.77%, 12/84), and the difference between the two groups was statistically significant (x2 = 51.862, *P* < 0.001). See Fig. 1.

### Risk factors influencing the occurrence of neurologic invasion in patients with PDAC

**Fig. 2:**
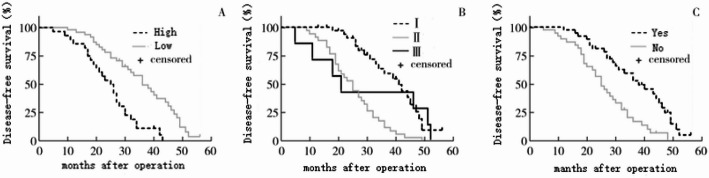
OS curves of PDAC patients with different clinicopathologic features. A: LAMA3 expression level; B:TNM staging; C:vascular invasion

To investigate the risk factors affecting the occurrence of nerve invasion in PDAC patients, Cox proportional analysis regression model was constructed. The results showed that LAMA3 expression level was an independent risk factor affecting the occurrence of nerve invasion in PDAC patients. See Table 2. Survival analysis showed that the median OS of patients with high LAMA3 expression and the occurrence of vascular invasion was significantly lower than that of patients with low LAMA3 expression and the occurrence of no vascular invasion (*P* < 0.001); and in TNM staging, the median OS of patients with stage II was significantly lower than that of patients with stage I (*P* < 0.001). See Fig. 2.

**Table 1:** Baseline data of 94 PDAC patients [*n* (%),*‾ x ± s*]

Variant			Variant		
Distinguishing between the sexes	Male	61 (64.89)	Degree of tumor differentiation	High differentiation	3 (3.19)
	Women	33 (35.11)		middle ground	52 (55.32)
Age (years)		61.31 ± 1.99		Medium-low differentiation	12 (12.77)
BMI (kg/m^2^ )	>24	54 (57.45)		low polarization	27 (28.72)
	≤ 24	40 (42.55)	lymphatic node transfer	there are	45 (47.87)
CEA (ng/ml)	≤ 5	62 (65.96)		not have	49 (52.13)
	>5	32 (34.04)	vascular invasion	there are	37 (39.36)
CA199 (U/ml)	≤ 200	48 (51.06)		not have	57 (60.64)
	> 200 and ≤ 500	18 (19.15)	TNM staging	Phase I	38 (40.43)
	>500	28 (29.79)		Phase II	16 (17.02)
Tumor size (cm)	≤ 2	5 (5.32)		Phase III	19 (20.21)
	> 2 and ≤ 4	64 (68.09)		Phase IV	21 (22.34)
	>4	25 (26.59)	medical history	History of diabetes	38 (40.43)
Tumor location	head of the pancreas	50 (53.19)		smoking history	29 (30.85)
	tail of pancreas	44 (46.81)		drinking history	27 (28.72)

**Table 2:** Unifactorial and multifactorial analysis affecting the occurrence of neurological invasion in patients with PDAC

Variant	One-way analysis of variance			Multifactorial analysis		
	HR	95% CI	*P*-value	HR	95% CI	*P*-value
distinguishing between the sexes	1.202	0.721–2.004	0.477	1.265	0.630–2.539	0.505
(a person’s) age	1.001	0.973–1.029	0.901	0.996	0.961–1.031	0.850
BMI	0.959	0.891–1.031	0.264	0.969	0.885–1.062	0.513
Tumor location	0.936	0.578–1.513	0.788	1.314	0.587–2.939	0.504
Tumor size	1.447	0.901–2.324	0.124	0.934	0.487–1.791	0.838
lymphatic node transfer	1.418	0.874–1.401	0.132	1.036	0.585–1.834	0.899
TNM staging	2.187	1.298–3.686	0.003^**^	1.799	0.854–3.790	0.121
vascular invasion	1.467	0.903–2.384	0.124	0.853	0.401–1.815	0.680
CEA	1.461	0.890–1.401	0.132	1.472	0.816–2.655	0.196
CA199	0.957	0.720–1.271	0.764	0.832	0.594–1.166	0.287
LAMA3 expression level	1.8776	1.105–3.185	0.019^*^	2.325	0.863–1.111	0.043
drinking wine	0.871	0.490–1.549	0.640	0.687	0.284–1.661	0.405
cigarette smoking	1.063	0.634–1.783	0.814	1.303	0.584–2.906	0.516
History of diabetes	0.619	0.374–1.024	0.062	0.669	0.365–1.226	0.194
Degree of tumor differentiation	1.582	0.977–2.559	0.060	1.407	0.738–2.683	0.298
Infiltration of the common bile duct	0.939	0.555–1.588	0.817	0.825	0.368–1.845	0.639
Duodenal infiltration	1.112	0.676–1.829	0.673	1.168	0.602–2.267	0.643
neural infiltration	1.161	0.643–2.093	0.618	1.236	0.548–2.788	0.607

## Discussion

The pathogenesis of PDAC is complex, involving mutations in KRAS, TP53, SMAD4, and CDKN2A and abnormal activation of Ras-MAPK, PI3K-AKT, and TGF-β signaling pathways [16]– [17].The prognosis of PDAC is extremely poor, because of a lack of specific symptoms in the early stage, and local infiltration and distant metastasis often occur at the time of diagnosis. Neural invasion is a typical invasive pathway in PDAC, which significantly affects the prognosis of patients. Currently, the existing diagnostic methods for detecting nerve invasion in PDAC are characterized by low sensitivity and specificity, and high operational risk [18]– [19], and there is an urgent need for the development of more sensitive and specific early diagnostic markers and techniques to improve the early detection rate and therapeutic efficacy of PDAC.

LAMA3 is a key component of laminin-332 in the basement membrane, and its overexpression in the tumor microenvironment has been implicated in cancer cell infiltration and metastasis. Elevated expression of LAMA3 may increase the likelihood of perineural invasion (PNI) by enhancing the adhesion and invasive capacity of PDAC cells toward perineural tissues [20]– [21]. Interestingly, Sari et al. [22] reported that LAMA3 expression had predictive value for liver metastasis in PDAC, although no association with PNI was observed, suggesting that the biological functions of LAMA3 may vary depending on metastatic sites. Previous studies indicate that aberrant expression of LAMA3 can influence tumor behavior through activation of signaling pathways such as PI3K/AKT and MAPK, thereby modulating cell survival, proliferation, and migration [23]– [24]. In our study, immunohistochemistry revealed that high LAMA3 expression was independently associated with PNI in PDAC patients, supporting its potential role in neural invasion. Mechanistically, LAMA3 may facilitate cancer cell migration and invasion by altering extracellular matrix (ECM) composition and enhancing tumor–nerve interactions [25]. While our data do not provide direct functional validation, this hypothesis is supported by prior studies showing that LAMA3 contributes to ECM remodeling and tumor invasiveness [26]– [27]. Moreover, it is plausible that the role of LAMA3 changes dynamically with tumor progression: in early stages, it may promote local expansion via cell adhesion and tissue remodeling, whereas in advanced stages, it may facilitate distant metastasis and PNI through ECM degradation and remodeling [28]. Therefore, monitoring dynamic changes in LAMA3 expression could provide additional insight into PDAC progression and prognosis.In addition, previous reports have suggested that LAMA3 may interact with integrins to activate PI3K/AKT and MAPK signaling pathways, thereby promoting cancer cell survival, proliferation, and migration [29–31]. Although our study did not directly assess these mechanisms, these pathways may underlie the observed association between high LAMA3 expression and increased neuroinvasion. Further in vitro and in vivo studies are needed to validate these mechanistic links.

The results of this study showed that LAMA3 expression was significantly higher in cancer tissues compared with paracancerous tissues (*P* < 0.05), suggesting that LAMA3 may play an important role in PDAC progression. Previous studies have indicated that LAMA3 could influence perineural invasion (PNI) by regulating extracellular matrix (ECM) composition and affecting tumor cell adhesion and migration [32]– [33]. High expression of LAMA3 may enhance interactions between pancreatic cancer cells and nerve cells, thereby facilitating tumor dissemination along nerve bundles [34–36]. As a potential molecular marker, detection of LAMA3 expression through immunohistochemistry or quantitative PCR may provide valuable information for early diagnosis and pathological evaluation in PDAC. Our results also showed that LAMA3 expression was associated with TNM stage, suggesting that it could contribute to assessing tumor aggressiveness and prognosis. Importantly, LAMA3 has been proposed as a potential therapeutic target in other malignancies, where inhibition of its expression or function reduced tumor invasion and metastasis [37]– [38]. Although our study did not directly evaluate therapeutic interventions, these findings support the hypothesis that targeting LAMA3 might improve outcomes in PDAC. Finally, dynamic changes in LAMA3 expression may reflect alterations in the tumor microenvironment, offering insights into the mechanisms of PDAC progression and tumor–nerve interactions. Further mechanistic studies are needed to validate these associations and to clarify whether LAMA3 can serve as an effective therapeutic target.

It is worth noting that no significant associations were found between LAMA3 expression and some clinicopathological variables, indicating that the role of LAMA3 may vary across different patient subgroups. These negative results suggest that LAMA3 should not be considered a universal marker, but rather one that interacts with specific molecular or clinical contexts. This study has several limitations. First, it was a retrospective single-center analysis with a relatively small sample size. Second, the follow-up duration was limited to 5 months, which may not fully reflect long-term survival outcomes. Finally, functional experiments to validate the biological mechanisms of LAMA3 in PDAC and perineural invasion were not performed. Future multi-center, prospective studies with longer follow-up and mechanistic investigations are warranted. Beyond its tissue-level expression, LAMA3 also holds promise as a non-invasive biomarker. Future studies should investigate whether LAMA3 can be detected in serum or other body fluids, which would allow for less invasive and more dynamic monitoring of tumor biology. In addition, molecular imaging approaches targeting LAMA3 may provide new opportunities for real-time assessment of tumor aggressiveness and therapeutic response. Clinically, integrating LAMA3 into risk stratification models could improve patient subtyping, prognosis prediction, and treatment decision-making. These potential applications highlight the translational value of LAMA3 and warrant further investigation in prospective, multi-center studies.

In summary, LAMA3 expression level is an independent risk factor for the development of neuroinvasion in PDAC patients, and LAMA3 expression level, TNM staging are correlated with the prognosis of PDAC patients, which could potentially be used as a valuable biomarker for the occurrence, development, and prediction of prognosis of patients with PDAC, and also as a potential therapeutic target for PDAC patients.

## Data Availability

All data analyzed in this study are available on request from the authors.
